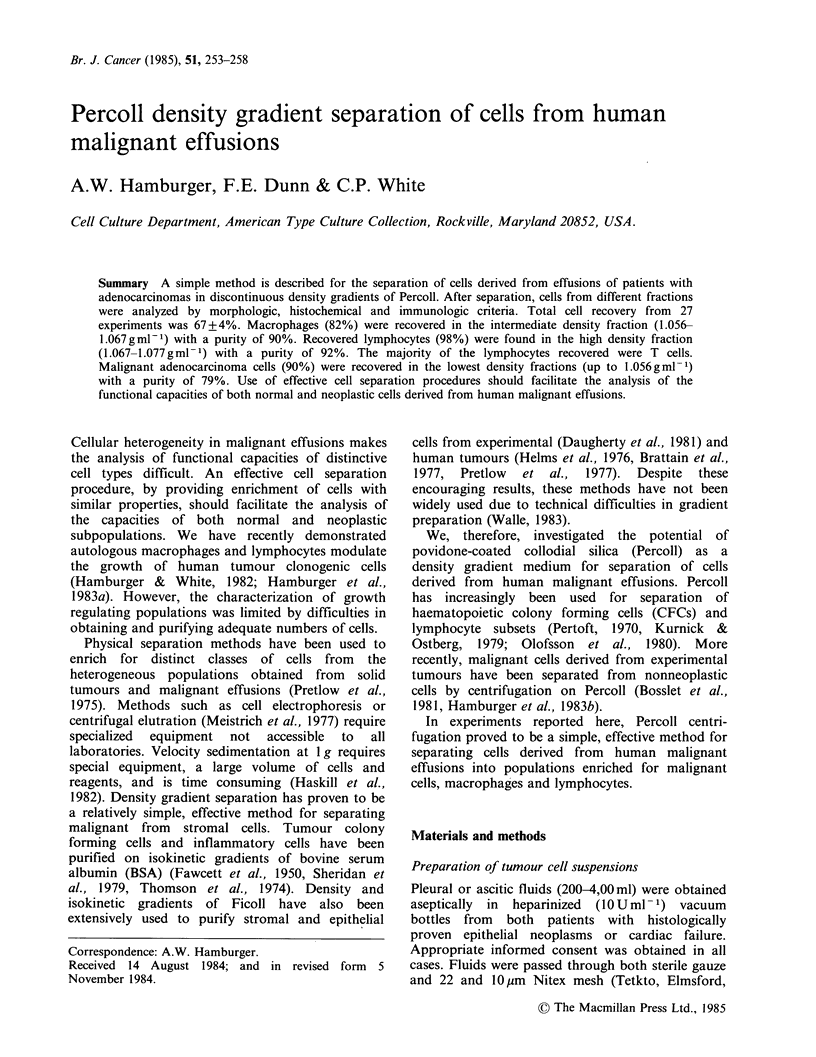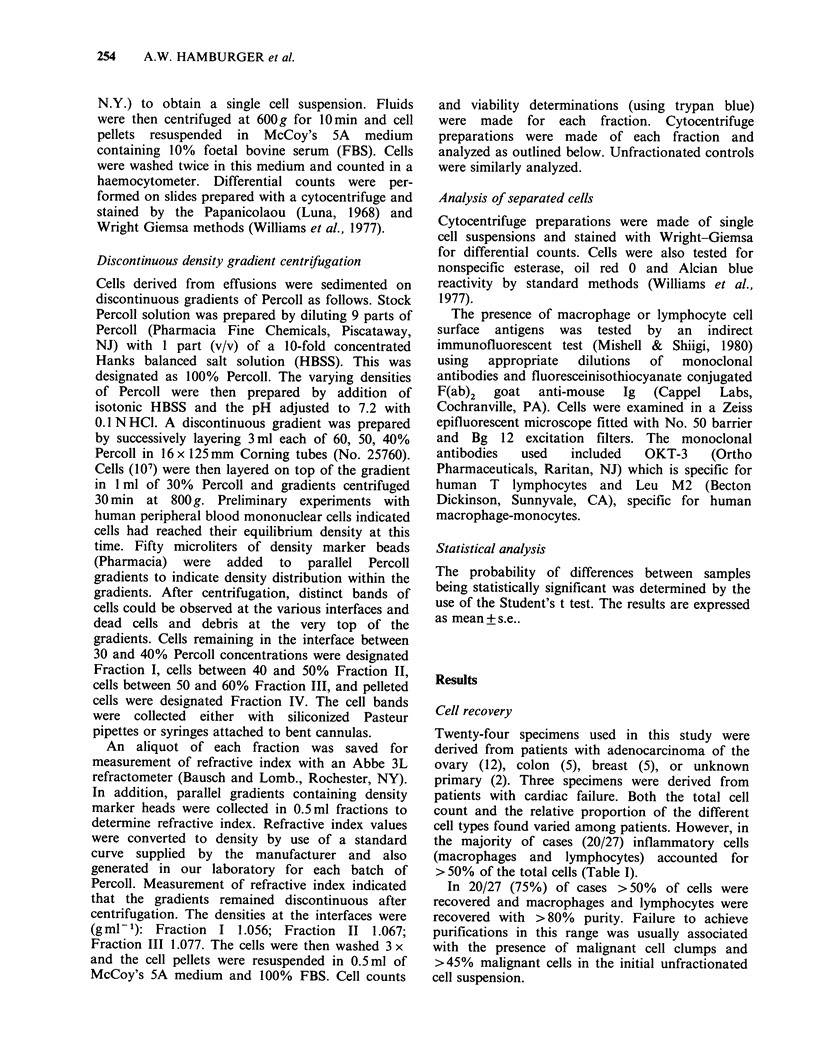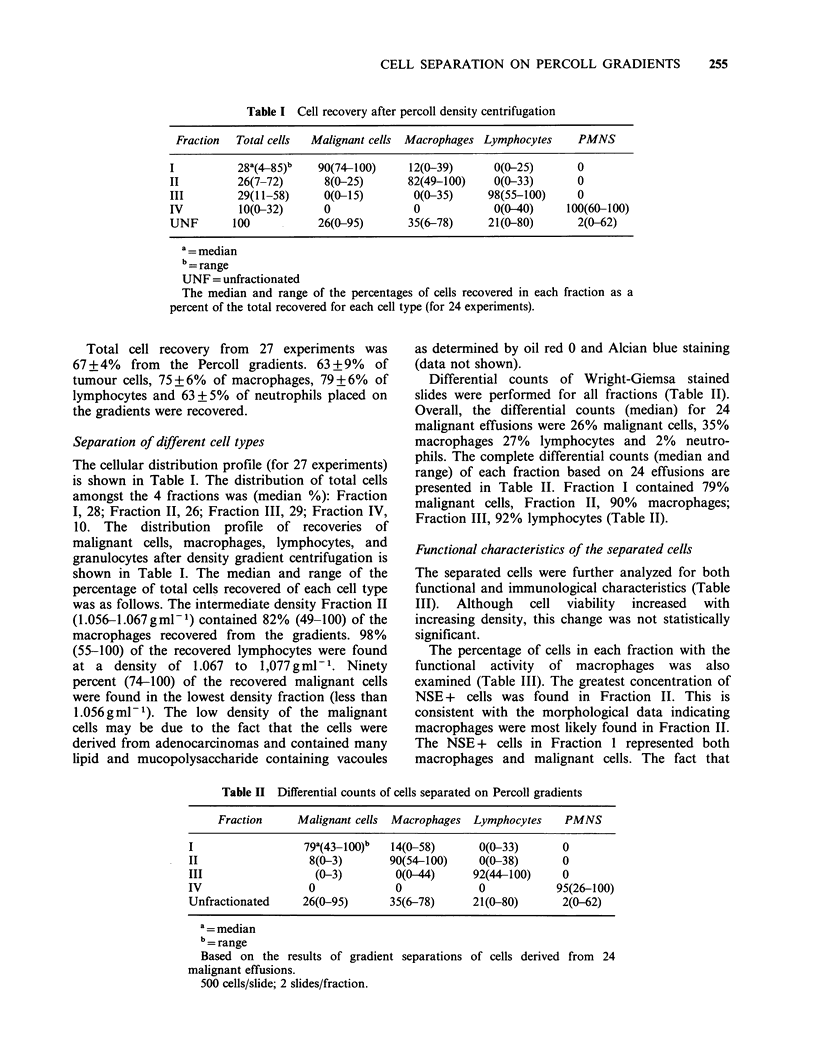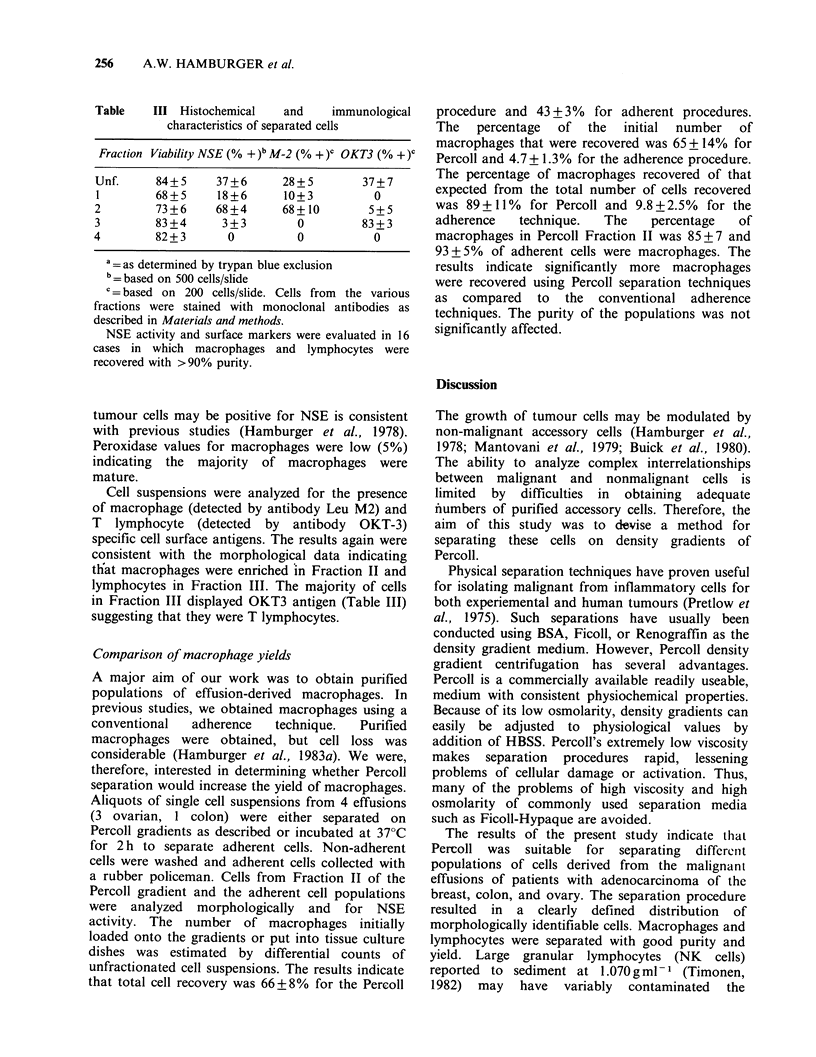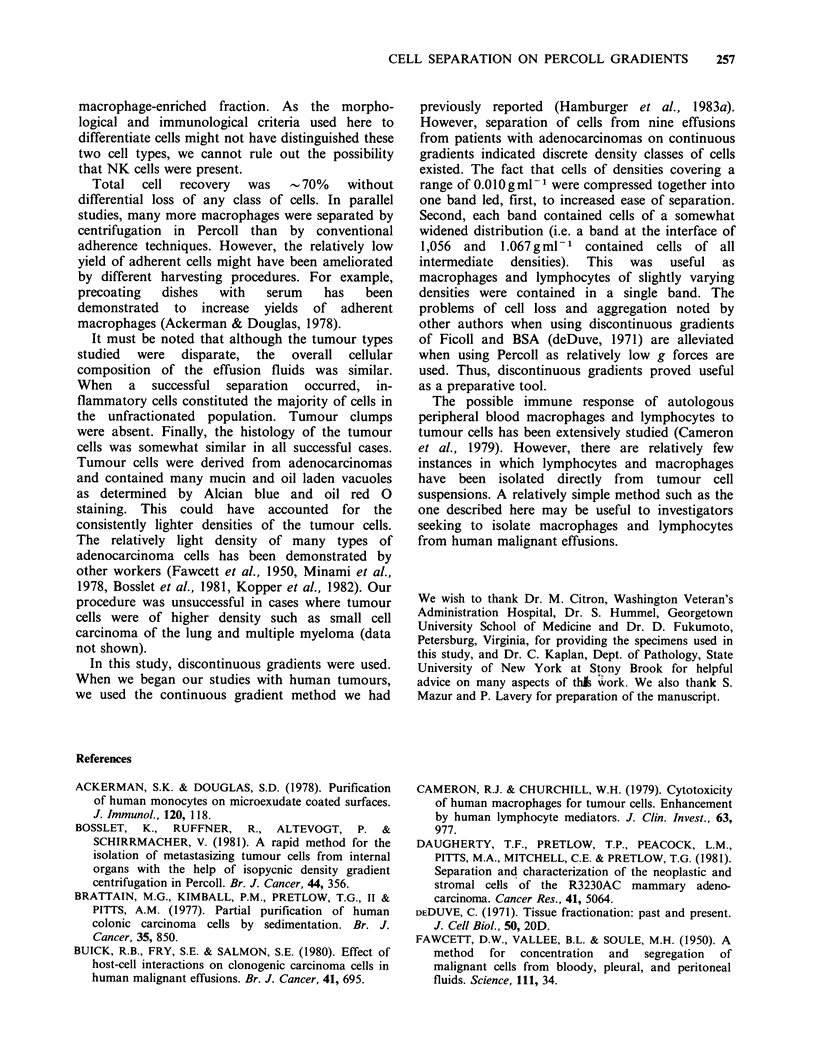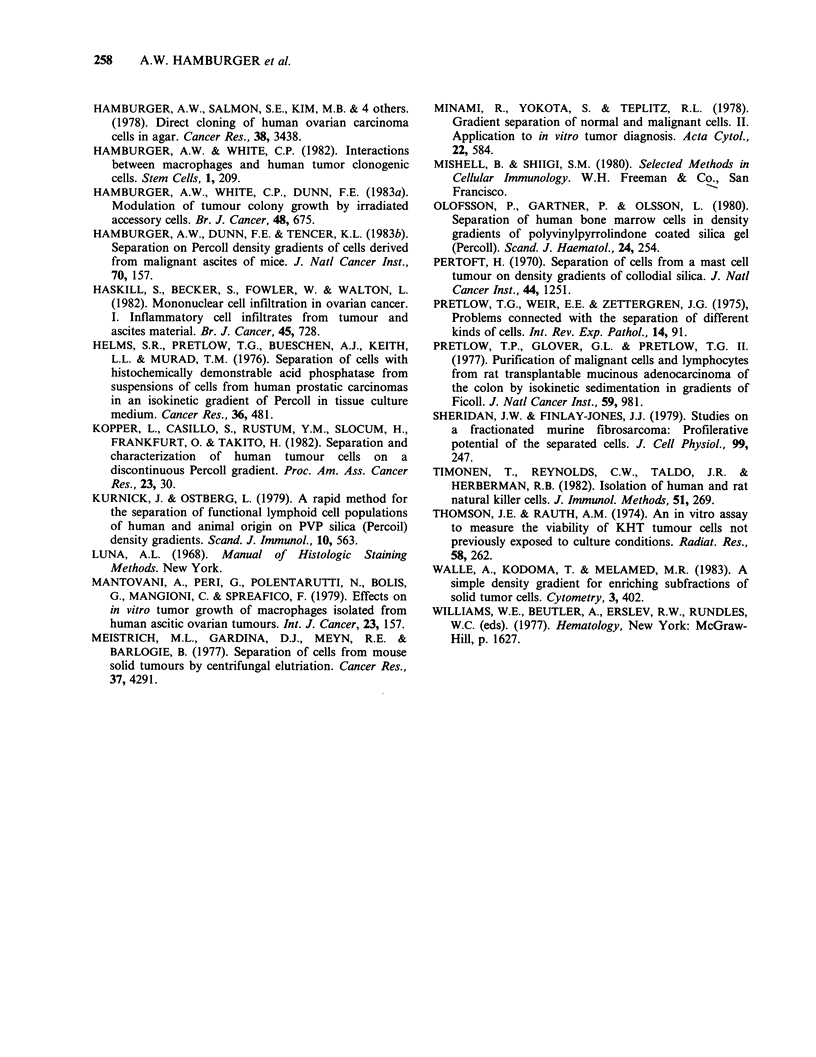# Percoll density gradient separation of cells from human malignant effusions.

**DOI:** 10.1038/bjc.1985.36

**Published:** 1985-02

**Authors:** A. W. Hamburger, F. E. Dunn, C. P. White

## Abstract

A simple method is described for the separation of cells derived from effusions of patients with adenocarcinomas in discontinuous density gradients of Percoll. After separation, cells from different fractions were analyzed by morphologic, histochemical and immunologic criteria. Total cell recovery from 27 experiments was 67 +/- 4%. Macrophages (82%) were recovered in the intermediate density fraction (1.056-1.067 g ml-1) with a purity of 90%. Recovered lymphocytes (98%) were found in the high density fraction (1.067-1.077 g ml-1) with a purity of 92%. The majority of the lymphocytes recovered were T cells. Malignant adenocarcinoma cells (90%) were recovered in the lowest density fractions (up to 1.056 g ml-1) with a purity of 79%. Use of effective cell separation procedures should facilitate the analysis of the functional capacities of both normal and neoplastic cells derived from human malignant effusions.


*Br. J. Cancer* (1985), **51**, 253–258

### Percoll density gradient separation of cells from human malignant effusions

A.W. Hamburger, F.E. Dunn & C.P. White

*Cell Culture Department, American Type Culture Collection, Rockville, Maryland 20852, USA.*

**Summary** A simple method is described for the separation of cells derived from effusions of patients with adenocarcinomas in discontinuous density gradients of Percoll. After separation, cells from different fractions were analyzed by morphologic, histochemical and immunologic criteria. Total cell recovery from 27 experiments was $67 \pm 4\%$. Macrophages (82%) were recovered in the intermediate density fraction (1.056–1.067 $g\,ml^{-1}$) with a purity of 90%. Recovered lymphocytes (98%) were found in the high density fraction (1.067–1.077 $g\,ml^{-1}$) with a purity of 92%. The majority of the lymphocytes recovered were T cells. Malignant adenocarcinoma cells (90%) were recovered in the lowest density fractions (up to 1.056 $g\,ml^{-1}$) with a purity of 79%. Use of effective cell separation procedures should facilitate the analysis of the functional capacities of both normal and neoplastic cells derived from human malignant effusions.

Cellular heterogeneity in malignant effusions makes the analysis of functional capacities of distinctive cell types difficult. An effective cell separation procedure, by providing enrichment of cells with similar properties, should facilitate the analysis of the capacities of both normal and neoplastic subpopulations. We have recently demonstrated autologous macrophages and lymphocytes modulate the growth of human tumour clonogenic cells (Hamburger & White, 1982; Hamburger *et al.*, 1983*a*). However, the characterization of growth regulating populations was limited by difficulties in obtaining and purifying adequate numbers of cells.

Physical separation methods have been used to enrich for distinct classes of cells from the heterogeneous populations obtained from solid tumours and malignant effusions (Pretlow *et al.*, 1975). Methods such as cell electrophoresis or centrifugal elutration (Meistrich *et al.*, 1977) require specialized equipment not accessible to all laboratories. Velocity sedimentation at 1 *g* requires special equipment, a large volume of cells and reagents, and is time consuming (Haskill *et al.*, 1982). Density gradient separation has proven to be a relatively simple, effective method for separating malignant from stromal cells. Tumour colony forming cells and inflammatory cells have been purified on isokinetic gradients of bovine serum albumin (BSA) (Fawcett *et al.*, 1950, Sheridan *et al.*, 1979, Thomson *et al.*, 1974). Density and isokinetic gradients of Ficoll have also been extensively used to purify stromal and epithelial cells from experimental (Daugherty *et al.*, 1981) and human tumours (Helms *et al.*, 1976, Brattain *et al.*, 1977, Pretlow *et al.*, 1977). Despite these encouraging results, these methods have not been widely used due to technical difficulties in gradient preparation (Walle, 1983).

We, therefore, investigated the potential of povidone-coated collodial silica (Percoll) as a density gradient medium for separation of cells derived from human malignant effusions. Percoll has increasingly been used for separation of haematopoietic colony forming cells (CFCs) and lymphocyte subsets (Pertoft, 1970, Kurnick & Ostberg, 1979; Olofsson *et al.*, 1980). More recently, malignant cells derived from experimental tumours have been separated from nonneoplastic cells by centrifugation on Percoll (Bosslet *et al.*, 1981, Hamburger *et al.*, 1983*b*).

In experiments reported here, Percoll centrifugation proved to be a simple, effective method for separating cells derived from human malignant effusions into populations enriched for malignant cells, macrophages and lymphocytes.

### Materials and methods

### *Preparation of tumour cell suspensions*

Pleural or ascitic fluids (200–4,00 ml) were obtained aseptically in heparinized (10 $U\,ml^{-1}$) vacuum bottles from both patients with histologically proven epithelial neoplasms or cardiac failure. Appropriate informed consent was obtained in all cases. Fluids were passed through both sterile gauze and 22 and 10 $\mu m$ Nitex mesh (Tetkto, Elmsford,

Correspondence: A.W. Hamburger.
Received 14 August 1984; and in revised form 5 November 1984.

© The Macmillan Press Ltd., 1985

N.Y.) to obtain a single cell suspension. Fluids were then centrifuged at 600*g* for 10 min and cell pellets resuspended in McCoy's 5A medium containing 10% foetal bovine serum (FBS). Cells were washed twice in this medium and counted in a haemocytometer. Differential counts were performed on slides prepared with a cytocentrifuge and stained by the Papanicolaou (Luna, 1968) and Wright Giemsa methods (Williams *et al.*, 1977).

*Discontinuous density gradient centrifugation*

Cells derived from effusions were sedimented on discontinuous gradients of Percoll as follows. Stock Percoll solution was prepared by diluting 9 parts of Percoll (Pharmacia Fine Chemicals, Piscataway, NJ) with 1 part (v/v) of a 10-fold concentrated Hanks balanced salt solution (HBSS). This was designated as 100% Percoll. The varying densities of Percoll were then prepared by addition of isotonic HBSS and the pH adjusted to 7.2 with 0.1 N HCl. A discontinuous gradient was prepared by successively layering 3 ml each of 60, 50, 40% Percoll in 16 x 125 mm Corning tubes (No. 25760). Cells ($10^7$) were then layered on top of the gradient in 1 ml of 30% Percoll and gradients centrifuged 30 min at 800*g*. Preliminary experiments with human peripheral blood mononuclear cells indicated cells had reached their equilibrium density at this time. Fifty microliters of density marker beads (Pharmacia) were added to parallel Percoll gradients to indicate density distribution within the gradients. After centrifugation, distinct bands of cells could be observed at the various interfaces and dead cells and debris at the very top of the gradients. Cells remaining in the interface between 30 and 40% Percoll concentrations were designated Fraction I, cells between 40 and 50% Fraction II, cells between 50 and 60% Fraction III, and pelleted cells were designated Fraction IV. The cell bands were collected either with siliconized Pasteur pipettes or syringes attached to bent cannulas.

An aliquot of each fraction was saved for measurement of refractive index with an Abbe 3L refractometer (Bausch and Lomb., Rochester, NY). In addition, parallel gradients containing density marker heads were collected in 0.5 ml fractions to determine refractive index. Refractive index values were converted to density by use of a standard curve supplied by the manufacturer and also generated in our laboratory for each batch of Percoll. Measurement of refractive index indicated that the gradients remained discontinuous after centrifugation. The densities at the interfaces were ($g\,ml^{-1}$): Fraction I 1.056; Fraction II 1.067; Fraction III 1.077. The cells were then washed 3 x and the cell pellets were resuspended in 0.5 ml of McCoy's 5A medium and 100% FBS. Cell counts and viability determinations (using trypan blue) were made for each fraction. Cytocentrifuge preparations were made of each fraction and analyzed as outlined below. Unfractionated controls were similarly analyzed.

*Analysis of separated cells*

Cytocentrifuge preparations were made of single cell suspensions and stained with Wright–Giemsa for differential counts. Cells were also tested for nonspecific esterase, oil red 0 and Alcian blue reactivity by standard methods (Williams *et al.*, 1977).

The presence of macrophage or lymphocyte cell surface antigens was tested by an indirect immunofluorescent test (Mishell & Shiigi, 1980) using appropriate dilutions of monoclonal antibodies and fluoresceinisothiocyanate conjugated $F(ab)_2$ goat anti-mouse Ig (Cappel Labs, Cochranville, PA). Cells were examined in a Zeiss epifluorescent microscope fitted with No. 50 barrier and Bg 12 excitation filters. The monoclonal antibodies used included OKT-3 (Ortho Pharmaceuticals, Raritan, NJ) which is specific for human T lymphocytes and Leu M2 (Becton Dickinson, Sunnyvale, CA), specific for human macrophage-monocytes.

*Statistical analysis*

The probability of differences between samples being statistically significant was determined by the use of the Student's t test. The results are expressed as mean ± s.e..

### Results

*Cell recovery*

Twenty-four specimens used in this study were derived from patients with adenocarcinoma of the ovary (12), colon (5), breast (5), or unknown primary (2). Three specimens were derived from patients with cardiac failure. Both the total cell count and the relative proportion of the different cell types found varied among patients. However, in the majority of cases (20/27) inflammatory cells (macrophages and lymphocytes) accounted for >50% of the total cells (Table I).

In 20/27 (75%) of cases >50% of cells were recovered and macrophages and lymphocytes were recovered with >80% purity. Failure to achieve purifications in this range was usually associated with the presence of malignant cell clumps and >45% malignant cells in the initial unfractionated cell suspension.

**Table I** Cell recovery after percoll density centrifugation

| *Fraction* | *Total cells* | *Malignant cells* | *Macrophages* | *Lymphocytes* | *PMNS* |
|---|---|---|---|---|---|
| I | 28[a](4–85)[b] | 90(74–100) | 12(0–39) | 0(0–25) | 0 |
| II | 26(7–72) | 8(0–25) | 82(49–100) | 0(0–33) | 0 |
| III | 29(11–58) | 0(0–15) | 0(0–35) | 98(55–100) | 0 |
| IV | 10(0–32) | 0 | 0 | 0(0–40) | 100(60–100) |
| UNF | 100 | 26(0–95) | 35(6–78) | 21(0–80) | 2(0–62) |

[a] = median
[b] = range
UNF = unfractionated
The median and range of the percentages of cells recovered in each fraction as a percent of the total recovered for each cell type (for 24 experiments).

Total cell recovery from 27 experiments was 67±4% from the Percoll gradients. 63±9% of tumour cells, 75±6% of macrophages, 79±6% of lymphocytes and 63±5% of neutrophils placed on the gradients were recovered.

### *Separation of different cell types*

The cellular distribution profile (for 27 experiments) is shown in Table I. The distribution of total cells amongst the 4 fractions was (median %): Fraction I, 28; Fraction II, 26; Fraction III, 29; Fraction IV, 10. The distribution profile of recoveries of malignant cells, macrophages, lymphocytes, and granulocytes after density gradient centrifugation is shown in Table I. The median and range of the percentage of total cells recovered of each cell type was as follows. The intermediate density Fraction II (1.056–1.067 $g ml^{-1}$) contained 82% (49–100) of the macrophages recovered from the gradients. 98% (55–100) of the recovered lymphocytes were found at a density of 1.067 to 1,077 $g ml^{-1}$. Ninety percent (74–100) of the recovered malignant cells were found in the lowest density fraction (less than 1.056 $g ml^{-1}$). The low density of the malignant cells may be due to the fact that the cells were derived from adenocarcinomas and contained many lipid and mucopolysaccharide containing vacoules as determined by oil red 0 and Alcian blue staining (data not shown).

Differential counts of Wright-Giemsa stained slides were performed for all fractions (Table II). Overall, the differential counts (median) for 24 malignant effusions were 26% malignant cells, 35% macrophages 27% lymphocytes and 2% neutrophils. The complete differential counts (median and range) of each fraction based on 24 effusions are presented in Table II. Fraction I contained 79% malignant cells, Fraction II, 90% macrophages; Fraction III, 92% lymphocytes (Table II).

### *Functional characteristics of the separated cells*

The separated cells were further analyzed for both functional and immunological characteristics (Table III). Although cell viability increased with increasing density, this change was not statistically significant.

The percentage of cells in each fraction with the functional activity of macrophages was also examined (Table III). The greatest concentration of NSE+ cells was found in Fraction II. This is consistent with the morphological data indicating macrophages were most likely found in Fraction II. The NSE+ cells in Fraction 1 represented both macrophages and malignant cells. The fact that

**Table II** Differential counts of cells separated on Percoll gradients

| *Fraction* | *Malignant cells* | *Macrophages* | *Lymphocytes* | *PMNS* |
|---|---|---|---|---|
| I | 79[a](43–100)[b] | 14(0–58) | 0(0–33) | 0 |
| II | 8(0–3) | 90(54–100) | 0(0–38) | 0 |
| III | (0–3) | 0(0–44) | 92(44–100) | 0 |
| IV | 0 | 0 | 0 | 95(26–100) |
| Unfractionated | 26(0–95) | 35(6–78) | 21(0–80) | 2(0–62) |

[a] = median
[b] = range
Based on the results of gradient separations of cells derived from 24 malignant effusions.
500 cells/slide; 2 slides/fraction.

**Table III** Histochemical and immunological characteristics of separated cells

| *Fraction* | *Viability* | *NSE* (% +)[b] | *M-2* (% +)[c] | *OKT3* (% +)[c] |
|---|---|---|---|---|
| Unf. | 84±5 | 37±6 | 28±5 | 37±7 |
| 1 | 68±5 | 18±6 | 10±3 | 0 |
| 2 | 73±6 | 68±4 | 68±10 | 5±5 |
| 3 | 83±4 | 3±3 | 0 | 83±3 |
| 4 | 82±3 | 0 | 0 | 0 |

[a] = as determined by trypan blue exclusion
[b] = based on 500 cells/slide
[c] = based on 200 cells/slide. Cells from the various fractions were stained with monoclonal antibodies as described in *Materials and methods*.

NSE activity and surface markers were evaluated in 16 cases in which macrophages and lymphocytes were recovered with >90% purity.

tumour cells may be positive for NSE is consistent with previous studies (Hamburger *et al.*, 1978). Peroxidase values for macrophages were low (5%) indicating the majority of macrophages were mature.

Cell suspensions were analyzed for the presence of macrophage (detected by antibody Leu M2) and T lymphocyte (detected by antibody OKT-3) specific cell surface antigens. The results again were consistent with the morphological data indicating that macrophages were enriched in Fraction II and lymphocytes in Fraction III. The majority of cells in Fraction III displayed OKT3 antigen (Table III) suggesting that they were T lymphocytes.

*Comparison of macrophage yields*

A major aim of our work was to obtain purified populations of effusion-derived macrophages. In previous studies, we obtained macrophages using a conventional adherence technique. Purified macrophages were obtained, but cell loss was considerable (Hamburger *et al.*, 1983*a*). We were, therefore, interested in determining whether Percoll separation would increase the yield of macrophages. Aliquots of single cell suspensions from 4 effusions (3 ovarian, 1 colon) were either separated on Percoll gradients as described or incubated at 37°C for 2h to separate adherent cells. Non-adherent cells were washed and adherent cells collected with a rubber policeman. Cells from Fraction II of the Percoll gradient and the adherent cell populations were analyzed morphologically and for NSE activity. The number of macrophages initially loaded onto the gradients or put into tissue culture dishes was estimated by differential counts of unfractionated cell suspensions. The results indicate that total cell recovery was 66±8% for the Percoll procedure and 43±3% for adherent procedures. The percentage of the initial number of macrophages that were recovered was 65±14% for Percoll and 4.7±1.3% for the adherence procedure. The percentage of macrophages recovered of that expected from the total number of cells recovered was 89±11% for Percoll and 9.8±2.5% for the adherence technique. The percentage of macrophages in Percoll Fraction II was 85±7 and 93±5% of adherent cells were macrophages. The results indicate significantly more macrophages were recovered using Percoll separation techniques as compared to the conventional adherence techniques. The purity of the populations was not significantly affected.

### Discussion

The growth of tumour cells may be modulated by non-malignant accessory cells (Hamburger *et al.*, 1978; Mantovani *et al.*, 1979; Buick *et al.*, 1980). The ability to analyze complex interrelationships between malignant and nonmalignant cells is limited by difficulties in obtaining adequate numbers of purified accessory cells. Therefore, the aim of this study was to devise a method for separating these cells on density gradients of Percoll.

Physical separation techniques have proven useful for isolating malignant from inflammatory cells for both experiemental and human tumours (Pretlow *et al.*, 1975). Such separations have usually been conducted using BSA, Ficoll, or Renograffin as the density gradient medium. However, Percoll density gradient centrifugation has several advantages. Percoll is a commercially available readily useable, medium with consistent physiochemical properties. Because of its low osmolarity, density gradients can easily be adjusted to physiological values by addition of HBSS. Percoll's extremely low viscosity makes separation procedures rapid, lessening problems of cellular damage or activation. Thus, many of the problems of high viscosity and high osmolarity of commonly used separation media such as Ficoll-Hypaque are avoided.

The results of the present study indicate that Percoll was suitable for separating different populations of cells derived from the malignant effusions of patients with adenocarcinoma of the breast, colon, and ovary. The separation procedure resulted in a clearly defined distribution of morphologically identifiable cells. Macrophages and lymphocytes were separated with good purity and yield. Large granular lymphocytes (NK cells) reported to sediment at 1.070 g ml$^{-1}$ (Timonen, 1982) may have variably contaminated the macrophage-enriched fraction. As the morphological and immunological criteria used here to differentiate cells might not have distinguished these two cell types, we cannot rule out the possibility that NK cells were present.

Total cell recovery was ~70% without differential loss of any class of cells. In parallel studies, many more macrophages were separated by centrifugation in Percoll than by conventional adherence techniques. However, the relatively low yield of adherent cells might have been ameliorated by different harvesting procedures. For example, precoating dishes with serum has been demonstrated to increase yields of adherent macrophages (Ackerman & Douglas, 1978).

It must be noted that although the tumour types studied were disparate, the overall cellular composition of the effusion fluids was similar. When a successful separation occurred, inflammatory cells constituted the majority of cells in the unfractionated population. Tumour clumps were absent. Finally, the histology of the tumour cells was somewhat similar in all successful cases. Tumour cells were derived from adenocarcinomas and contained many mucin and oil laden vacuoles as determined by Alcian blue and oil red O staining. This could have accounted for the consistently lighter densities of the tumour cells. The relatively light density of many types of adenocarcinoma cells has been demonstrated by other workers (Fawcett *et al.*, 1950, Minami *et al.*, 1978, Bosslet *et al.*, 1981, Kopper *et al.*, 1982). Our procedure was unsuccessful in cases where tumour cells were of higher density such as small cell carcinoma of the lung and multiple myeloma (data not shown).

In this study, discontinuous gradients were used. When we began our studies with human tumours, we used the continuous gradient method we had previously reported (Hamburger *et al.*, 1983*a*). However, separation of cells from nine effusions from patients with adenocarcinomas on continuous gradients indicated discrete density classes of cells existed. The fact that cells of densities covering a range of 0.010 g ml$^{-1}$ were compressed together into one band led, first, to increased ease of separation. Second, each band contained cells of a somewhat widened distribution (i.e. a band at the interface of 1,056 and 1.067 g ml$^{-1}$ contained cells of all intermediate densities). This was useful as macrophages and lymphocytes of slightly varying densities were contained in a single band. The problems of cell loss and aggregation noted by other authors when using discontinuous gradients of Ficoll and BSA (deDuve, 1971) are alleviated when using Percoll as relatively low *g* forces are used. Thus, discontinuous gradients proved useful as a preparative tool.

The possible immune response of autologous peripheral blood macrophages and lymphocytes to tumour cells has been extensively studied (Cameron *et al.*, 1979). However, there are relatively few instances in which lymphocytes and macrophages have been isolated directly from tumour cell suspensions. A relatively simple method such as the one described here may be useful to investigators seeking to isolate macrophages and lymphocytes from human malignant effusions.

We wish to thank Dr. M. Citron, Washington Veteran's Administration Hospital, Dr. S. Hummel, Georgetown University School of Medicine and Dr. D. Fukumoto, Petersburg, Virginia, for providing the specimens used in this study, and Dr. C. Kaplan, Dept. of Pathology, State University of New York at Stony Brook for helpful advice on many aspects of this work. We also thank S. Mazur and P. Lavery for preparation of the manuscript.

**References**

ACKERMAN, S.K. & DOUGLAS, S.D. (1978). Purification of human monocytes on microexudate coated surfaces. *J. Immunol.*, **120,** 118.

BOSSLET, K., RUFFNER, R., ALTEVOGT, P. & SCHIRRMACHER, V. (1981). A rapid method for the isolation of metastasizing tumour cells from internal organs with the help of isopycnic density gradient centrifugation in Percoll. *Br. J. Cancer*, **44,** 356.

BRATTAIN, M.G., KIMBALL, P.M., PRETLOW, T.G., II & PITTS, A.M. (1977). Partial purification of human colonic carcinoma cells by sedimentation. *Br. J. Cancer*, **35,** 850.

BUICK, R.B., FRY, S.E. & SALMON, S.E. (1980). Effect of host-cell interactions on clonogenic carcinoma cells in human malignant effusions. *Br. J. Cancer*, **41,** 695.

CAMERON, R.J. & CHURCHILL, W.H. (1979). Cytotoxicity of human macrophages for tumour cells. Enhancement by human lymphocyte mediators. *J. Clin. Invest.*, **63,** 977.

DAUGHERTY, T.F., PRETLOW, T.P., PEACOCK, L.M., PITTS, M.A., MITCHELL, C.E. & PRETLOW, T.G. (1981). Separation and characterization of the neoplastic and stromal cells of the R3230AC mammary adenocarcinoma. *Cancer Res.*, **41,** 5064.

DEDUVE, C. (1971). Tissue fractionation: past and present. *J. Cell Biol.*, **50,** 20D.

FAWCETT, D.W., VALLEE, B.L. & SOULE, M.H. (1950). A method for concentration and segregation of malignant cells from bloody, pleural, and peritoneal fluids. *Science*, **111,** 34.

HAMBURGER, A.W., SALMON, S.E., KIM, M.B. & 4 others. (1978). Direct cloning of human ovarian carcinoma cells in agar. *Cancer Res.*, **38**, 3438.

HAMBURGER, A.W. & WHITE, C.P. (1982). Interactions between macrophages and human tumor clonogenic cells. *Stem Cells*, **1**, 209.

HAMBURGER, A.W., WHITE, C.P., DUNN, F.E. (1983*a*). Modulation of tumour colony growth by irradiated accessory cells. *Br. J. Cancer*, **48**, 675.

HAMBURGER, A.W., DUNN, F.E. & TENCER, K.L. (1983*b*). Separation on Percoll density gradients of cells derived from malignant ascites of mice. *J. Natl Cancer Inst.*, **70**, 157.

HASKILL, S., BECKER, S., FOWLER, W. & WALTON, L. (1982). Mononuclear cell infiltration in ovarian cancer. I. Inflammatory cell infiltrates from tumour and ascites material. *Br. J. Cancer*, **45**, 728.

HELMS, S.R., PRETLOW, T.G., BUESCHEN, A.J., KEITH, L.L. & MURAD, T.M. (1976). Separation of cells with histochemically demonstrable acid phosphatase from suspensions of cells from human prostatic carcinomas in an isokinetic gradient of Percoll in tissue culture medium. *Cancer Res.*, **36**, 481.

KOPPER, L., CASILLO, S., RUSTUM, Y.M., SLOCUM, H., FRANKFURT, O. & TAKITO, H. (1982). Separation and characterization of human tumour cells on a discontinuous Percoll gradient. *Proc. Am. Ass. Cancer Res.*, **23**, 30.

KURNICK, J. & OSTBERG, L. (1979). A rapid method for the separation of functional lymphoid cell populations of human and animal origin on PVP silica (Percoil) density gradients. *Scand. J. Immunol.*, **10**, 563.

LUNA, A.L. (1968). *Manual of Histologic Staining Methods*. New York.

MANTOVANI, A., PERI, G., POLENTARUTTI, N., BOLIS, G., MANGIONI, C. & SPREAFICO, F. (1979). Effects on *in vitro* tumor growth of macrophages isolated from human ascitic ovarian tumours. *Int. J. Cancer*, **23**, 157.

MEISTRICH, M.L., GARDINA, D.J., MEYN, R.E. & BARLOGIE, B. (1977). Separation of cells from mouse solid tumours by centrifungal elutriation. *Cancer Res.*, **37**, 4291.

MINAMI, R., YOKOTA, S. & TEPLITZ, R.L. (1978). Gradient separation of normal and malignant cells. II. Application to *in vitro* tumor diagnosis. *Acta Cytol.*, **22**, 584.

MISHELL, B. & SHIIGI, S.M. (1980). *Selected Methods in Cellular Immunology*. W.H. Freeman & Co., San Francisco.

OLOFSSON, P., GARTNER, P. & OLSSON, L. (1980). Separation of human bone marrow cells in density gradients of polyvinylpyrrolindone coated silica gel (Percoll). *Scand. J. Haematol.*, **24**, 254.

PERTOFT, H. (1970). Separation of cells from a mast cell tumour on density gradients of collodial silica. *J. Natl Cancer Inst.*, **44**, 1251.

PRETLOW, T.G., WEIR, E.E. & ZETTERGREN, J.G. (1975), Problems connected with the separation of different kinds of cells. *Int. Rev. Exp. Pathol.*, **14**, 91.

PRETLOW, T.P., GLOVER, G.L. & PRETLOW, T.G. II. (1977). Purification of malignant cells and lymphocytes from rat transplantable mucinous adenocarcinoma of the colon by isokinetic sedimentation in gradients of Ficoll. *J. Natl Cancer Inst.*, **59**, 981.

SHERIDAN, J.W. & FINLAY-JONES, J.J. (1979). Studies on a fractionated murine fibrosarcoma: Profilerative potential of the separated cells. *J. Cell Physiol.*, **99**, 247.

TIMONEN, T., REYNOLDS, C.W., TALDO, J.R. & HERBERMAN, R.B. (1982). Isolation of human and rat natural killer cells. *J. Immunol. Methods*, **51**, 269.

THOMSON, J.E. & RAUTH, A.M. (1974). An in vitro assay to measure the viability of KHT tumour cells not previously exposed to culture conditions. *Radiat. Res.*, **58**, 262.

WALLE, A., KODOMA, T. & MELAMED, M.R. (1983). A simple density gradient for enriching subfractions of solid tumor cells. *Cytometry*, **3**, 402.

WILLIAMS, W.E., BEUTLER, A., ERSLEV, R.W., RUNDLES, W.C. (eds). (1977). *Hematology*, New York: McGraw-Hill, p. 1627.